# Double-Strand Breaks Induce Nuclear-Cytosolic Shuttling of Polymorphic DNA Mismatch Repair Protein MutS Homolog 3 and Binding to NEMO/IKKγ in Colon Cancer Cells

**DOI:** 10.1016/j.gastha.2025.100756

**Published:** 2025-07-25

**Authors:** Stephanie S. Tseng-Rogenski, Minoru Koi, John M. Carethers

**Affiliations:** 1 Department of Internal Medicine, University of Michigan, Ann Arbor, Michigan; 2 Division of Gastroenterology and Hepatology, Department of Medicine, University of California San Diego, San Diego, California; 3 Moores Cancer Center, University of California San Diego, San Diego, California; 4 Herbert Wertheim School of Public Health and Longevity Science, University of California San Diego, San Diego, California

**Keywords:** MSH3, mismatch repair, NFκB, NEMO, IKKγ, colorectal cancer, homologous recombination, DNA double strand breaks, interleukin-6, etoposide, microsatellite instability, reactive oxygen species, oxidative stress

## Abstract

**Background and Aims:**

Human MutS homolog 3 (MSH3) is a DNA mismatch repair protein that recognizes postsynthetic slippage mistakes at microsatellite sequences consisting of dinucleotide or longer repeats in concert with its heterodimer partner MSH2. MSH3 has also been implicated in DNA double-strand break (DSB) repair as part of Homologous Recombination. Loss of MSH3 function is an acquired somatic defect seen in 50% of colorectal cancers and triggered by proinflammatory interleukin-6 signaling, causing a reversible nuclear-to-cytoplasmic shift of the protein. With MSH3’s shift to the cytosol, microsatellite frameshift mutations accumulate. Here we examined MSH3 and Δ27bpMSH3, an MSH3 polymorph that alters function of MSH3’s nuclear localization signal with enhancement for cytosolic retention with interleukin-6 and oxidative stress, for evidence of dysfunction with induced DSBs.

**Methods:**

We employed immunofluorescent microscopy, nuclear-cytosolic protein fractionation, immunoprecipitation and quantitative reverse transcriptase polymerase chain reaction to track the location and amount of MSH3 and Δ27bpMSH3 after inducing DSBs with etoposide.

**Results:**

Cells containing polymorphic Δ27bpMSH3 were more susceptible to induced DSBs, and induced DSBs triggered nuclear-to-cytosolic shift of Δ27bpMSH3 that involved binding to the NFκB protein NEMO/IKKγ.

**Conclusion:**

These findings suggest that inflammatory proteins are important for Δ27bpMSH3 translocation to the cytosol but also for stabilizing MSH3 when separated from nuclear MSH2.

## Introduction

DNA mismatch repair (MMR) utilizes 2 main complexes to detect postsynthetic mistakes after DNA replication, MutSα and MutSβ, that lead to repair or trigger cell death before mitosis.^1^, ^2^, ^3^, ^4^ MutSα, comprising human MutS homologs 2 (MSH2) and 6 (MSH6) recognizes single nucleotides mispairs and slippage mistakes at mononucleotide or dinucleotide repeats; MutSβ, comprising MSH2 and human MutS homolog 3 (MSH3) recognizes slippage mistakes at dinucleotide or longer repeats.^2^^,^^5^^,^^6^ After recognition, another MMR complex, MutLα, comprising of the human MutL homolog 1 (MLH1) and human postmitotic segregation homolog 2 (PMS2), binds to MutSα and MutSβ to effectualize repair signaling.^1^, ^2^, ^3^, ^4^, ^5^, ^6^ Germline mutations in *MSH2*, *MSH6*, *MLH1*, and *PMS2* (and *EPCAM*) are the cause of the hereditary cancer condition known as Lynch syndrome; biallelic somatic mutations in these MMR proteins cause a Lynch-like syndrome,^1^^,^^7^^,^^8^ and somatic hypermethylation of *MLH1* is responsible for the ∼15% of sporadic colorectal cancers that demonstrated microsatellite instability.^1^, ^2^, ^3^, ^4^^,^^9^ Interestingly, mutation of *MSH3* is not a contributory cause of Lynch syndrome, but biallelic germline mutations do cause an oligopolyposis syndrome among rare families.^10^^,^^11^ Specifically, MSH3 becomes dysfunctional in 50% of colorectal cancers by a somatic loss-of-function mechanism from proinflammatory interleukin-6 (IL6)/JAK/STAT3 signaling and/or oxidative stress via nuclear-to-cytosolic shifting of MSH3.^2^^,^^6^^,^^12^, ^13^, ^14^, ^15^ With isolated dysfunction of MSH3 due to a cytosolic location, cells demonstrate frameshifts at dinucleotide or longer DNA microsatellite sequences (termed elevated microsatellite alterations at selected tetranucleotide repeats or EMAST); given the relationship with the proinflammatory cytokine IL6, this MMR defect has also been described as inflammation-associated microsatellite alterations.^16^ This MSH3 defect also encompasses the etiology of microsatellite instability-low since essentially all frameshifted markers were dinucleotide frameshifts and not mononucleotide frameshifts in studies.^2^^,^^17^^,^^18^

Due to this observed ability to shift locations with IL6, we investigated, identified and mapped MSH3’s nuclear localization signal (NLS) and 2 nuclear export signals.^13^ We discovered that MSH3 had a polymorphic region proximal to its NLS heavily enriched in alanine and proline ([A]_12_PPAPPAPA), and a specific polymorph we termed *Δ27bpMSH3* (due to an imperfect deletion of three copies of an imperfect 9bp repeat in this region) altered the function of the NLS allowing Δ27bpMSH3 to more easily translocate and remain sequestered in the cytosol as compared to wild type (WT) MSH3.^13^ The *Δ27bpMSH3* genotype is present in some colorectal cancer cells lines (e.g. SW480^Δ27/Δ27^) and colon tissue samples from patients with ulcerative colitis, much higher than the general health population (0.0012%, UCSC database, https://genome.ucsc.edu),^13^ suggesting that Δ27bpMSH3 could be somatically selected for during inflammation and tumorigenesis. Persons with germline Δ27bpMSH3 in theory may be more prone to inflammatory-induced cancer.

Additionally, MSH3 is involved in double-strand break (DSB) repair through homologous recombination (HR), unique among the MMR proteins.^2^ HR is mostly active during S- and G2-phases when sister chromatids are available to serve as the template during the repair and is therefore faithful to the original sequences (unlike nonhomologous end-joining (NHEJ) repair that is more error-prone and used when there is not a sister chromatid available, and DNA-PKcs plays a critical role). During HR, DNA is cut near the lesion to create a single-stranded 3′ end, which is quickly covered by replication-binding protein. Recent studies have shown that MSH2/MSH3 are essential in stabilizing the free 3′-ends during HR.^19^, ^20^, ^21^, ^22^ At the later stage of repairing process, BRCA1/BRCA2/PALB2 and then RAD51 are recruited sequentially. RAD51 plays a key role in the actual repairing reaction, including homology searching, strand exchange, and Holliday junction formation.^23^^,^^24^ There is strong association between high sensitivity to a DNA-PKcs inhibitor, KU60648, and MSH3 deficiency (null or mutated), suggesting that without functional MSH3, cells are addicted to NHEJ repair, highlighting a mechanistic pathway to cell death through DNA-PKcs inhibitors.^25^ Interestingly, the MSH3 “in frame deletion” variants in this study that showed sensitivity are genotyped *Δ27bpMSH3*.^25^

Here we examined MSH3 and Δ27bpMSH3 for evidence of dysfunction with induced DSBs. Polymorphic Δ27bpMSH3 cells were more susceptible to induced DSBs, and induced DSBs triggered nuclear-to-cytosolic shift of Δ27bpMSH3 that involved binding to the NFκB protein NEMO/IKKγ.

## Materials and Methods

### Cell Lines and Reagents

CaCO2^WT/WT^ and SW480^Δ27/Δ27^ cells are purchased from ATCC. CaCO2^WT/WT^ was grown in DMEM and SW480^Δ27/Δ27^ was grown in IMDM. Both media was supplemented with 10% FBS and 1x penicillin-streptomycin. All cell culture reagents were from Invitrogen (Waltham, MA). Mouse anti-MLH1 (clone G168-728, catalog #554073), anti-MSH2 (catalog #556349), anti-MSH3 (catalog #611390), and anti-MSH6 (catalog #610918) antibodies were from BD Biosciences (San Jose, CA). Rabbit monoclonal anti-MSH3 (EPR4334(2), catalog #ab111107), anti-NEMO/IKKγ (EPR16629, catalog #ab178872) and antihistone H3 (catalog #ab18521) antibodies were from Abcam (Cambridge, UK). Mouse anti-α-tubulin (catalog #T6074), etoposide (catalog #341205), and Trichostatin A (catalog #T8552) were from Sigma-Aldrich (St. Louis, MO). The anti-γ-H2A.X antibody (RM224, catalog #MA-33062) and the reagents for detection of the ROS generation were from Invitrogen.

### IFM

Cells were seated onto the 4-well chamber slides (40,000 cells/well) and incubated at 37 °C/5% CO_2_ overnight. For etoposide treatment, cells were treated with 100 μM etoposide for the time indicated. Upon completion of treatment, cells were fixed with iced-cold acetone for 5 minutes, air dried, and stored at 4 °C until staining. Staining has been described previously.^6^^,^^12^^,^^13^

### Nuclear and Cytosolic Protein Fractionation

The fractionation kit was purchased from Rockland (Limerick, PA). To treat cells for fractionation experiments, equal number (2–3 million) of CaCO2^WT/WT^ and SW480^Δ27/Δ27^ cells were seeded onto each 10 cm dishes and incubated at 37 °C/5% CO_2_ overnight. Cells were treated with the indicated reagent before subjected to fractionation procedure. The methodology for the nuclear-cytosolic protein fractionation was documented in detail previously.^12^ Briefly, cells from one 10-cm dish were collected and resuspended in 5 ml of 1x Buffer A to wash and count cells. Cells were centrifuged at 300x g and then resuspended in 1x Buffer A to adjust cell density to 6.6 million cells per ml. Fifty μl of cells (330,000 cells per reaction) was used for each reaction. Fifty μl of freshly prepared Buffer B was added into each tube, and cells were incubated on a rotator at RT for 7 minute, followed by centrifugation at 5,000x g at 4 °C for 1 minute. The supernatant was collected, separately, and recentrifuged at 100,00x g at 4 °C for 1 minute. The supernatant was collected, which was the cytosolic fraction. The pellets from both centrifugations were pooled and resuspended in 50 μl of 1x Buffer A, to which 50 μl of freshly prepared Buffer C with Detergent II was added and centrifuged immediately at 5,000x g at 4 °C for 1 minute. The supernatant was collected and recentrifuged at 100,00x g at 4 °C for 1 minute. The pellets (nuclei) from the 2 centrifugations were pooled, individually, and resuspended in 100 μl of 1X Buffer A. Twenty-5 μl of 5X SDS-PAGE sample buffer was added into each cytosolic and/or nuclear fraction and heated at 60 °C for 10 minute. Equal volume of cytosolic and nuclear proteins was used for the subsequent Western-Blotting (WB) analysis.

### IP and WB

Cells were seeded and treated as described. Whole cell lysates using Native Protein Isolation Kit (Invitrogen) were used for IP reactions. Anti-rabbit IgG beads (50 μl per reaction; Rockland) were washed with 1x PBS, equilibrated with lysis buffer for at least 20 minutes on ice, and incubated with cell lysate on ice for 2–3 hours. Cell lysates were removed, and the beads were washed with 0.02% NP-40 in 1X PBS 4 times, 5 minutes each wash. Beads were heated with 50 μl of 2x SDS-PAGE sample buffer at 100 °C for 10 minutes, followed by centrifugation at 100,00x g for 5 minutes. The supernatants were collected for WB analysis as previously described.^12^ The Rabbit TrueBlot WB kit from Rockland Antibody and Assays was used to detect proteins after IP to avoid detecting the rabbit IgG heavy and light chain from the antibody used for IP reactions.

### Quantitative Reverse Transcriptase Polymerase Chain Reaction

RNA was isolated using the RNA Isolation Kit (Qiagen; Hilden, Germany) and quantified. Two μg of RNA per sample was used to set up reverse transcription reactions using iScript Advanced Reverse Transcription Kit (Bio-Rad; Hercules, CA). The primers used in the quantitative polymerase chain reaction were purchased from Bio-Rad. The controls used were CHMP2A and VCP. The SYBER Green reaction mix for the quantitative reverse transcriptase polymerase chain reaction was from Bio-Rad.

### Detection of ROS

The assay kit was purchased from Invitrogen and assays were performed following company’s instructions. Briefly, cells were treated with 100 μM etoposide overnight. The next day, assay substrate was added to the cells and incubated for 1 hour in the incubator. Cells were collected and subjected to Flow-Cytometry Core at the University of Michigan to measure ROS generation upon each treatment.

### Statistical Analyses

We used student T-test (2-tailed; different variation, Microsoft Excel), with significance set at the 0.05 level.

## Results

### Δ27bpMSH3 Cells are More Susceptible to Growth Inhibition with DSB-Inducing Etoposide than WT MSH3 Cells

**Figure 1:**
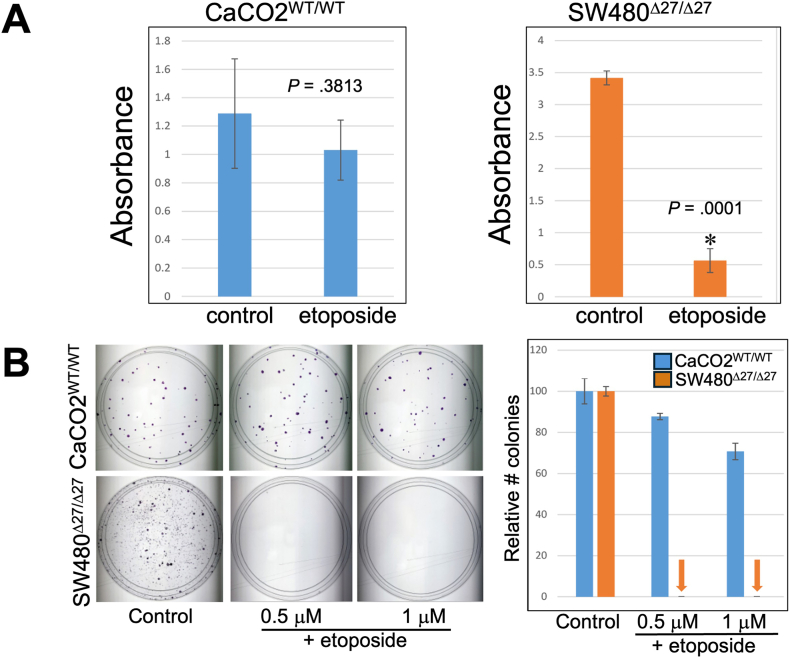
SW480^Δ27/Δ27^ cells are significantly more sensitive to growth inhibition with etoposide than CaCO2^WT/WT^ cells. (A) After 100 μM etoposide treatment, SW480^Δ27/Δ27^ cells show markedly slowed cell growth compared to CaCO2^WT/WT^ cells and (B) an inability to form colonies with just 0.5 μM or 1 μM etoposide.

It was previously reported that human cancer cells containing null or *Δ27bpMSH3* alleles were deficient in DSB repair through HR when the NHEJ pathway was pharmacologically inhibited by KU60648.^25^ Because we previously showed that Δ27bpMSH3 has a greater tendency to exit the nucleus and accumulate in the cytoplasm compared to the WT MSH3 with oxidative stress or IL6,^13^ we investigated if Δ27bpMSH3 plays a role in the DSB repair deficiency via HR. We first tested the sensitivity to KU60648 of CaCO2^WT/WT^ and SW480^Δ27/Δ27^ cells. SW480^Δ27/Δ27^ cells were not more sensitive to KU60648 or another DNA-PK inhibitor than CaCO2^WT/WT^ (Figure A1A). Since both cell lines express MLH1, this result is consistent with previous findings that MLH1 is associated with NHEJ pathway ^26^ and that loss of MLH1 can sensitize colon cancer cells to KU60648.^27^ An association between MSH3 and NHEJ pathway has not been previously reported, and these results are consistent with no involvement. We then combined treatment of the DNA-PK inhibitor with the DSB-inducer etoposide at various doses. We observed that SW480^Δ27/Δ27^ compared to CaCO2^WT/WT^ cells were highly susceptible to etoposide. Addition of the DNA-PK inhibitor did not have detectable additional effects (Figure A1B), indicating that there was observable difference in HR but not NHEJ between these 2 MSH3 polymorphic cell lines. Henceforth, we focused on etoposide for this study (100 μM etoposide for 18–24 hours). We discovered that SW480^Δ27/Δ27^ cells grew significantly slower than CaCO2^WT/WT^ after etoposide (Figure 1A). Furthermore, SW480^Δ27/Δ27^ cells completely lost ability to form colonies after etoposide treatment, in sharp contrast to CaCO2^WT/WT^ cells (Figure 1B). These results suggest that Δ27bpMSH3 may be less functional for HR compared to WT MSH3 after DSBs.

### Etoposide Treatment Triggers Δ27bpMSH3 Nuclear-To-Cytosolic Shift in Absence of and Independent from Proinflammatory Stimuli, with Reduced Rad51 Foci than WT MSH3 Cells

**Figure 2:**
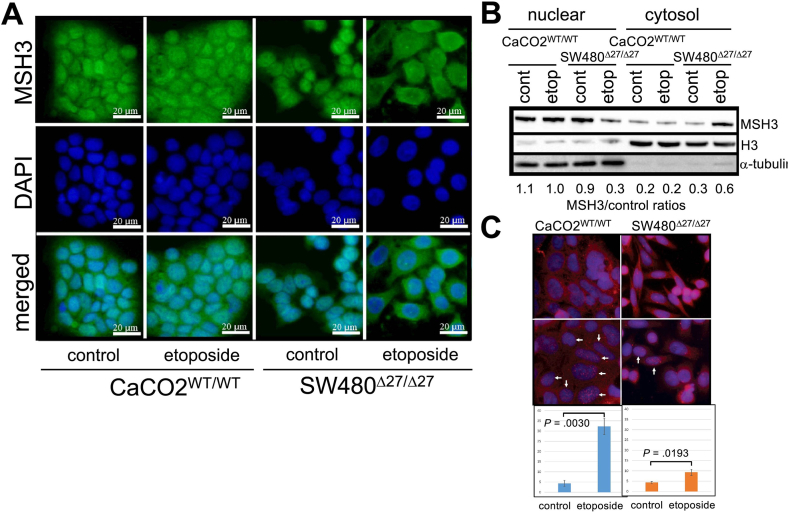
Δ27bpMSH3 accumulates in the cytosol after etoposide treatment in SW480^Δ27/Δ27^ cells, while WT MSH3 remains in the nucleus in CaCO2^WT/WT^ cells. (A) IFM was performed to examine the subcellular localization of MSH3. CaCO2^WT/WT^ and SW480^Δ27/Δ27^ cells were treated with 100 μM etoposide overnight (18 hours). Δ27bpMSH3 in SW480^Δ27/Δ27^ cells could be observed to empty out from the nucleus and accumulate in the cytosol after etoposide treatment, while WT MSH3 in CaCO2^WT/WT^ remained in the nucleus. (B) Nuclear-cytosolic protein fractionation confirming results from the IFM experiments and showing the accumulation of Δ27bpMSH3 in the cytosol in SW480^Δ27/Δ27^ cells after etoposide treatment. (C) Rad51 foci are efficiently detected in CaCO2^WT/WT^ after etoposide treatment but are less common in SW480^Δ27/Δ27^ cells. Cells were treated with 100 μM etoposide for 18 hr and immunostained for Rad51 to quantitate numbers of cells containing more than 50 large Rad51 foci. There was a 6-fold increase of Rad51 foci in CaCO2^WT/WT^ cells, while only a 2-fold increase is evident in SW480^Δ27/Δ27^ cells. For this experiment, we evaluated 100 cells in each of triplicate experiments (300 cells evaluated in total).

We initially followed the treatment protocol described in Dietlein *et al*^25^ where cells are treated with 10 μM etoposide for 1 hour followed by removal of etoposide, allowing cells to recover (4–24 hours). Under these conditions, we did not detect any Δ27bpMSH3 cytosolic accumulation by immunofluorescent microscopy (IFM), even with higher dosage of etoposide. We then allowed etoposide treatment to go longer (2–24 hours) and observed that Δ27bpMSH3 accumulated in the cytoplasm of SW480^Δ27/Δ27^ cells with longer treatment (18–28 hours), but not WT MSH3 in CaCO2^WT/WT^ cells (Figure 2AB). Dietlein *et al.* reported that null- or Δ27bpMSH3-expressing cells failed to recruit Rad51 to repair DSBs induced by etoposide when NHEJ was blocked.^25^ Similarly, under our longer treatment experimental protocol, we observed significantly more Rad51 foci in CaCO2^WT/WT^ than in SW480^Δ27/Δ27^ cells after etoposide (Figure 2C). Because Rad51-foci can only form in S/G2/M cell cycle phase, we checked expression of Germinin, a S/G2/M marker. Upon etoposide treatment, CaCO2^WT/WT^ but not SW480^Δ27/Δ27^ cells showed significant upregulation of Germinin (Figure A2), indicating that G1 arrest could be a reason for fewer Rad51-foci formation in SW480^Δ27/Δ27^ cells. Taken together, our data indicate that DSB repair in CaCO2^WT/WT^ cells is functional while DSB repair in SW480^Δ27/Δ27^ cells are impaired.

### WT MSH3 and Δ27bpMSH3 Bind NEMO/IKKγ, Part of the Complex that Activates NFκB, in the Nucleus

**Figure 3:**
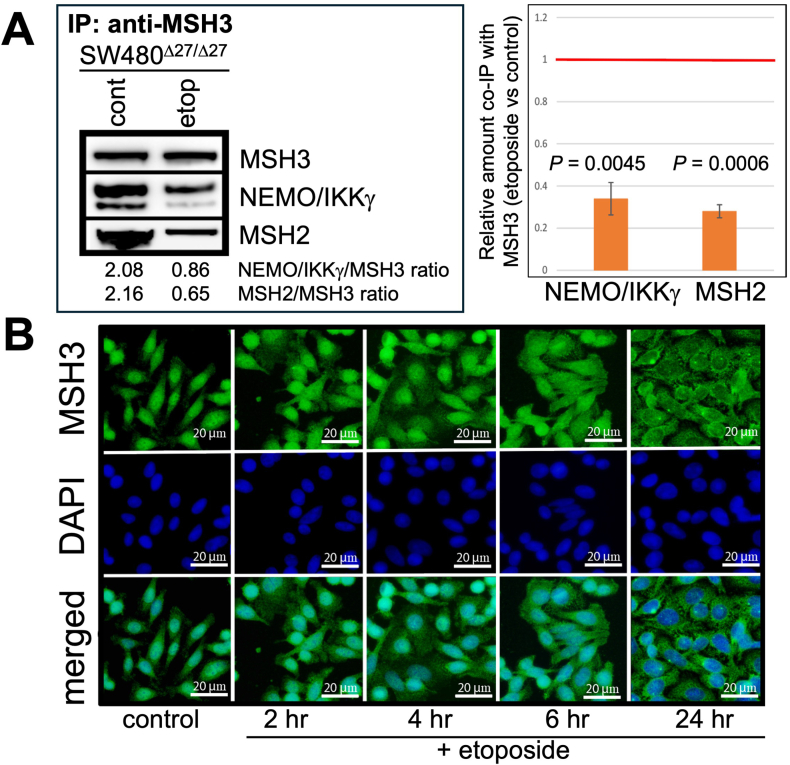
Δ27bpMSH3 binds to NEMO/IKKγ as well as MSH2 and dissociates from both NEMO/IKKγ and MSH2 upon etoposide treatment to accumulate in the cytosol. (A) SW480^Δ27/Δ27^ cells were treated with 100 μM etoposide continuously for 18 hours. Anti-MSH3 IP experiments revealed interaction between Δ27bpMSH3 and NEMO/IKKγ (as well as between Δ27bpMSH3 and MSH2), with both interactions being reduced after etoposide treatment. This experiment was repeated three times for statistical analyses. (B) SW480^Δ27/Δ27^ cells were treated with etoposide for the times indicated. Immunofluorescent microscopy revealed that with longer etoposide treatment there is diminished nuclear Δ27bpMSH3 and increased cytosolic Δ27bpMSH3.

NEMO/IKKγ is the regulatory subunit of the IKK complex that activates NFκB. It shuttles between the nucleus and cytoplasm as a part of DNA damage response.^28^ Considering both WT MSH3, Δ27bpMSH3, and NEMO are capable of shuttle between the nucleus and cytoplasm, we examined if MSH3 or Δ27bpMSH3 and NEMO/IKKγ exit the nucleus as a complex upon etoposide treatment. We first treated CaCO2^WT/WT^ and SW480^Δ27/Δ27^ cells with etoposide to monitor DSBs at different time points. We observed drastic increase of γ-H2A.X expression indicating induced DSBs as soon as 2 hours after treatment that lasted for at least 24 hours (Figure A3AB). We co-immunoprecipitated both MSH2 and NEMO/IKKγ with Δ27bpMSH3 in SW480^Δ27/Δ27^ cells using MSH3 antibodies (Figure 3A) indicating an interaction between its known heterodimer partner MSH2 as well the novel finding of an interaction with NEMO/IKKγ. With etoposide treatment, levels of Δ27bpMSH3-NEMO/IKKγ and Δ27bpMSH3-MSH2 both decrease (Figure 3A). Because Δ27bpMSH3 in SW480^Δ27/Δ27^ was observed to accumulate in the cytoplasm (Figure 2AB), we conducted a time-course study to closely monitor the localization of Δ27bpMSH3 in response to etoposide. As treatment time increased, less Δ27bpMSH3 was detected in the nucleus and simultaneously more cytosolic Δ27bpMSH3 were observed in SW480^Δ27/Δ27^ cells (Figure 3B). We did not observe changes of the staining patterns regarding the nuclear-cytosolic NEMO/IKKγ distribution by IFM (Figure A4) initially suggesting discordance between the location of these 2 interacting proteins after induced DSBs.

**Figure 4:**
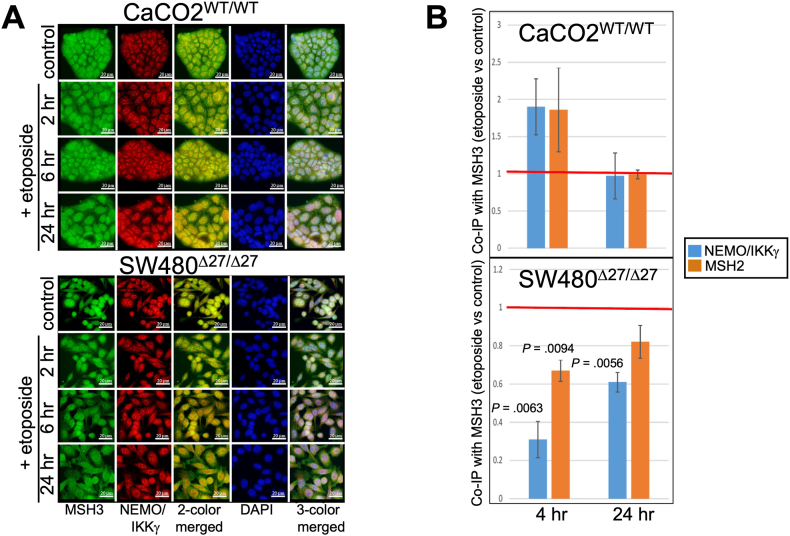
Δ27bpMSH3 and MSH3 colocalize and bind NEMO/IKKγ in the nucleus within 6 hours after etoposide treatment, with Δ27bpMSH3 dissociating from NEMO/IKKγ and accumulating in the cytosol thereafter. (A) Immunofluorescent microscopy using anti-MSH3 and anti-NEMO/IKKγ antibodies to examine cellular localization of proteins. WT MSH3 and NEMO colocalized in the nucleus and remained there through 24 hours after etoposide treatment in CaCO2^WT/WT^. On the contrary, Δ27bpMSH3 colocalized in the nucleus early then gradually exited the nucleus in SW480^Δ27/Δ27^ and accumulated in the cytosol while NEMO/IKKγ remained in the nucleus. (B) Anti-MSH3 IP experiments revealed increased interaction between WT MSH3 and both NEMO/IKKγ and MSH2 at 4 hours but diminished Δ27bpMSH3-NEMO/IKKγ and Δ27bpMSH3-MSH2 interactions. At 24 hours, the level of interactions was back to basal levels in CaCO2^WT/WT^, while the interactions remained reduced in SW480^Δ27/Δ27^, especially Δ27bpMSH3-NEMO/IKKγ.

We monitored the timeline of the Δ27bpMSH3 shift and NEMO interaction through IFM experiments. We observed that in our double-immunostaining experiments, MSH3 and NEMO/IKKγ colocalized between 2 and 6 hours post-treatment in both CaCO2^WT/WT^ and SW480^Δ27/Δ27^ cells within the nucleus. At 24 hours post-treatment there was still colocalization of MSH3 with NEMO/IKKγ in CaCO2^WT/WT^ in the nucleus, but not in SW480^Δ27/Δ27^ cells where NEMO was in the nucleus while Δ27bpMSH3 had moved to the cytoplasm (Figure 4A). Through immunoprecipitation (IP) experiments at the 4-hour time point, there was increased amount of both MSH2 and NEMO/IKKγ that interacted with MSH3 in CaCO2^WT/WT^, but both interactions decreased with Δ27bpMSH3 in SW480^Δ27/Δ27^ (Figure 4B). At 24 hours after etoposide treatment, levels of interaction were comparable between untreated and treated groups for both NEMO/IKKγ and MSH2 in CaCO2^WT/WT^. These interactions remained reduced in SW480^Δ27/Δ27^ compared to untreated cells, (Figure 4B). Examining the NEMO/IKKγ-MSH3 interaction closer within the first 5 hours after etoposide treatment, there was increase in CaCO2^WT/WT^ but a graduate decrease of Δ27bpMSH3- NEMO/IKKγ interaction in SW480^Δ27/Δ27^ (Figure A5).

**Figure 5:**
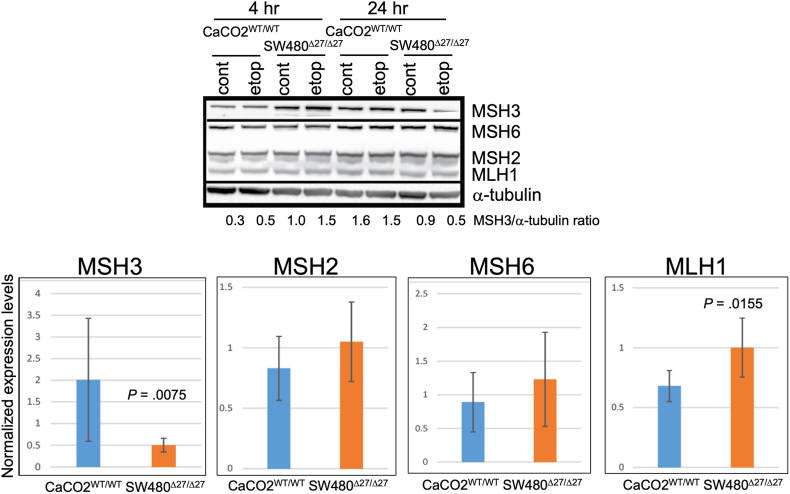
Steady-state levels of Δ27bpMSH3 protein decrease after etoposide treatment in SW480^Δ27/Δ27^ cells with no significant effect on other mismatch repair proteins or WT MSH3 in CaCO2^WT/WT^ cells. (*Upper panel*) Western blots assessing MMR protein expression in cells treated with 100 μM etoposide for 4 and 24 hours. Note in CaCO2^WT/WT^ cells that WT MSH3 is upregulated at 4 hours then returns to the level of untreated control cells at 24 hours. In SW480^Δ27/Δ27^ cells the amount of Δ27bpMSH3 also increased at 4 hours but decreased at 24 hours. Other MMR proteins remained unchanged. (*Lower panels*) Quantification of MMR protein extracts normalized to α-tubulin after 100 μM etoposide treatment for 28 hours. Shown are fold-change after etoposide vs untreated control. The amount of Δ27bpMSH3 in SW480^Δ27/Δ27^ cells decreased significantly (*P* = .0075) in sharp contrast to other MMR proteins and WT MSH3 in CaCO2^WT/WT^ cells. The decrease of MLH1 in CaCO2^WT/WT^ cells was significant (*P* = .0155). Statistics were calculated from 4 biological repeats of experiments.

**Figure 6:**
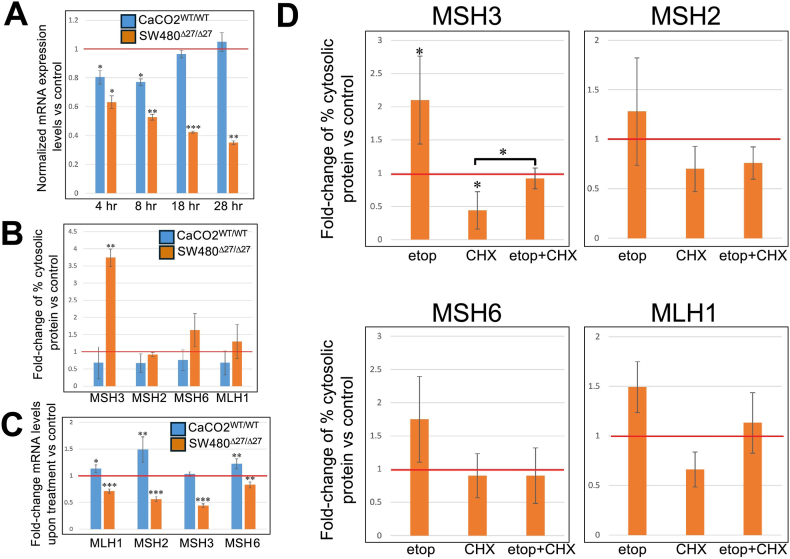
(A) Etoposide treatment causes a time-dependent reduction in *Δ27bpMSH3* mRNA but not WT *MSH3* mRNA. Measuring mRNA via RT-PCR after etoposide treatment, WT MSH3 mRNA in CaCO2^WT/WT^ cells were reduced slightly initially (*P* = .0188 for 4 hours and 0.0034 for 8 hours) but returned to untreated control levels thereafter. On the contrary, Δ27bpMSH3 mRNA in SW480^Δ27/Δ27^ cells decreased in a time-dependent manner post-treatment (*P* = .0045 for 4 hours; *P* = .0006 for 8 hours; *P* < .0001 for 18 hours; *P* = .0001 for 28 hours). (B) Δ27bpMSH3 protein moves from the nucleus to the cytosol while depressing *Δ27bpMSH3* and other MMR mRNA levels with etoposide treatment. Fold-change of percentage of MMR proteins after nuclear-cytosolic fractionation and WB comparing 100 μM etoposide for 18-hour vs untreated controls. Note significant cytosolic shift of Δ27bpMSH3 in SW480^Δ27/Δ27^ cells post-treatment that was absent from WT MSH3 in CaCO2^WT/WT^ cells. Statistics were calculated from three repeats of experiments. (C) Fold-change of MMR mRNA measured by RT-PCR after 18-hour etoposide treatment compared to untreated controls. Etoposide-treated CaCO2^WT/WT^ cells expressed more *MLH1*, *MSH2*, and *MSH6* (*P* = .0148 for *MLH1*, 0.0100 for *MSH2*, and 0.0064 for *MSH6*). On the contrary, SW480^Δ27/Δ27^ cells downregulated all MMR mRNA (*P* < .0001 for *MLH1*, *MSH2*, and *MSH3*; *P* = .0026 for *MSH6*). Statistics were calculated based on 5 sets of repeat experiments. (D) Both the pre-existing and newly synthesized Δ27bpMSH3 protein contribute to the cytosolic Δ27bpMSH3 pool with etoposide treatment. SW480^Δ27/Δ27^ cells were treated with etoposide (Etop), CHX, or both compounds before nuclear-cytosolic fractionation to quantitate percentages of cytosolic MMR proteins. Etoposide alone increased cytosolic Δ27bpMSH3 (*P* = .0449 vs control) and cycloheximide alone reduced cytosolic Δ27bpMSH3 (*P* = .0277 vs control); combined etoposide and cycloheximide treatment increased Δ27bpMSH3 compared to cycloheximide alone (*P* = .0316 vs cycloheximide alone). This indicates that the cytosolic Δ27bpMSH3 pool observed in etoposide alone is composed of pre-existing Δ27bpMSH3 protein that shifts from the nucleus as well as newly synthesized Δ27bpMSH3 protein. CHX, cycloheximide. RT-PCR, reverse transcriptase polymerase chain reaction.

We also assessed all MMR proteins expression levels 4 hours and 24 hours after etoposide treatment. Interestingly, the amount of MSH3 increased at 4 hours post-treatment in both cell lines, suggesting overexpressing or stabilization of MSH3. At 24 hours post-treatment, MSH3 expression level remained the same in CaCO2^WT/WT^ but decreased in SW480^Δ27/Δ27^ cells while expression levels of all other MMR proteins remained unchanged (Figure 5, *top*). With quantitation, the amount of Δ27bpMSH3 in SW480^Δ27/Δ27^ cells were markedly decreased, while levels of other MMR proteins were not significantly affected by treatment except for MLH1 in CaCO2^WT/WT^ cells (Figure 5, *bottom*). These observed patterns for MSH3 and MSH2 upon treatment were confirmed using another antibody (Figure A6). With etoposide treatment, Δ27bpMSH3 mRNA in SW480^Δ27/Δ27^ demonstrated a time-dependent reduction (Figure 6A), suggesting Δ27bpMSH3 expression is being degraded or its expression is being shut down. This is in contrast to MSH3 in CaCO2^WT/WT^ where there was slight reduction initially (at 4 and 8 hours) that returned to normal levels at later time points (18 and 28 hours). Overall, our results suggest that after etoposide treatment, WT MSH3 is fully available to engage with DNA repair processes and its interaction with NEMO in the nucleus whereas Δ27bpMSH3 is not due to its exit from the nucleus and level of mRNA production.

### Significant Fraction of Δ27bpMSH3 is Detected in the Cytoplasm after Induced DSBs from Both Nuclear Shift of Existing Protein and New Protein Synthesis

We performed nuclear-cytosolic protein fractionation to quantitate MMR proteins and their location in the cell. Within SW480^Δ27/Δ27^ cells, we observed a significant increase in cytosolic Δ27bpMSH3 while the distribution of other MMR proteins did not markedly change with treatment (Figure 6B). Within CaCO2^WT/WT^ cells, all MMR proteins, including WT MSH3, appeared to increase their nuclear amounts (with decreased percentages of cytosolic proteins), although the changes did not reach statistical significance (Figure 6B). These patterns were confirmed by another antibody (Figure A7). In response to etoposide, MMR mRNA levels were depressed in SW480^Δ27/Δ27^ cells but stable to elevated in CaCO2^WT/WT^ cells (Figure 6C). Our results confirm that SW480^Δ27/Δ27^ cells after etoposide treatment have greatly diminished nuclear Δ27bpMSH3 and are therefore deficient in MSH3-associated DNA repair.

We evaluated if the observed cytosolic Δ27bpMSH3 is derived from the nuclear Δ27bpMSH3 that shuttles into the cytoplasm in response to etoposide and DSBs or is newly synthesized protein. We blocked protein synthesis with cycloheximide in SW480^Δ27/Δ27^ cells to suppress *de novo* protein synthesis in addition to etoposide treatment. Nuclear-cytosolic fractionation showed an increase of cytosolic Δ27bpMSH3 in the double-treatment group compared to cycloheximide alone, with no significant changes for MSH2, MSH6, and MLH1 (Figure 6D), indicating that nuclear Δ27bpMSH3 indeed exited the nucleus and relocated to the cytoplasm, contributing to the cytosolic Δ27bpMSH3 pool along with the newly synthesized protein upon etoposide treatment. This distribution pattern of Δ27bpMSH3 and MSH2 were confirmed using different antibodies (Figure A8). There was more cytosolic Δ27bpMSH3 with etoposide treatment alone, demonstrating that some newly synthesized Δ27bpMSH3 also contributed to the cytosolic Δ27bpMSH3 pool. Overall, both existing nuclear and newly synthesized Δ27bpMSH3 contributes to the cytosolic pool after induced DSBs.

### Δ27bpMSH3 Protein Levels are Affected by Proteosome-Associated Degradation

**Figure 7:**
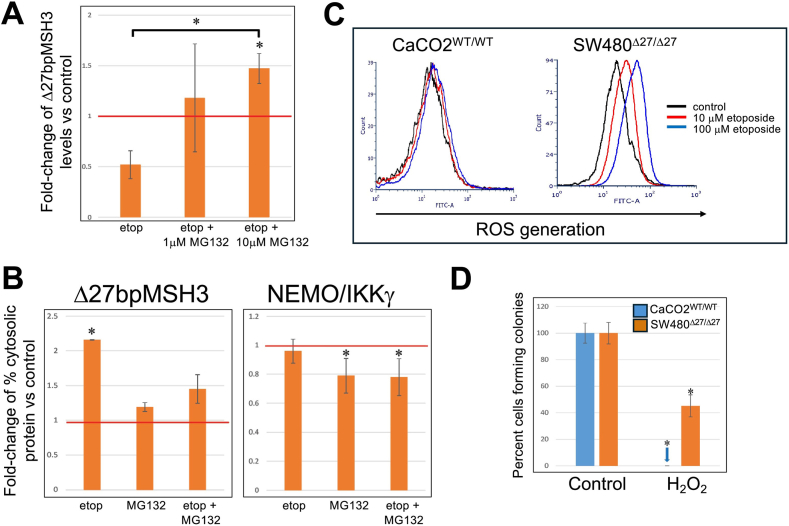
(A) Inhibition of proteasome-associated protein degradation rescues Δ27bpMSH3 protein levels and modifies the nuclear-cytosolic distribution of Δ27bpMSH3 and NEMO/IKKγ after etoposide treatment. Total Δ27bpMSH3 protein levels in whole lysates from SW480^Δ27/Δ27^ cells. Etoposide (Etop) treatment alone reduced Δ27bpMSH3 (*P* = .0263 vs control), and addition of 1 μM MG132 to etoposide treatment restored Δ27bpMSH3 to the levels of the control (*P* = .6233 vs control). Addition of 10 μM of MG132 markedly increased Δ27bpMSH3 (*P* = .0317 vs control; *P* = .0013 vs etoposide). (B) Fold-change (FC) of percentage of cytosolic Δ27bpMSH3 and NEMO/IKKγ from SW480^Δ27/Δ27^ cells. MG132 alone did not impact distribution of Δ27bpMSH3 (*P* = .4365) but inclusion of MG132 with etoposide reduced the percentage of cytosolic Δ27bpMSH3 (*P* = .0048 for etoposide vs control; *P* = .2005 for etoposide + MG132 vs control). Etoposide treatment alone did not impact nuclear-cytosolic distribution of NEMO/IKKγ (*P* = .4055 etoposide vs control). MG132 treatment alone decreased the percentage of cytosolic NEMO/IKKγ (ie increased percentage of nuclear NEMO/IKKγ) with or without etoposide (*P* = .0380 for MG132 vs control; *P* = .0416 etoposide + MG132 vs control). (C) Etoposide treatment of SW480^Δ27/Δ27^ cells trigger ROS generation that is absent from CaCO2^WT/WT^ cells. Flow cytometry analyses show upregulation of ROS in etoposide treated SW480^Δ27/Δ27^ cells, but not in CaCO2^WT/WT^ cells. (D) SW480^Δ27/Δ27^ cells are significantly more resistant to growth inhibition with exogenous oxidative stress than CaCO2^WT/WT^ cells. Colony forming assays were utilized after cells were treated with 50 μM of H_2_O_2_. CaCO2^WT/WT^ cells could not form colonies that were large enough to be counted (*P* = .0005) while nearly 50% of SW480^Δ27/Δ27^ cells did (*P* = .0003).

Our time-course studies revealed that the amount of Δ27bpMSH3 mRNA in SW480^Δ27/Δ27^ cells decreased by 4 hours after etoposide with continued reduction thereafter (Figure 6A), followed by a decrease in Δ27bpMSH3 protein by 24–28 hours (Figure 5). Indeed, there was an increase in Δ27bpMSH3 protein at 4 hours post-treatment (Figure 5). These observations might indicate that Δ27bpMSH3 was being stabilized initially then degraded over time. To examine if proteasome-associated degradation played a role in decreasing Δ27bpMSH3 by 28 hours, we treated SW480^Δ27/Δ27^ cells with both etoposide and MG132 (a proteasome degradation inhibitor) to quantitate total Δ27bpMSH3 post-treatment. Addition of MG132 increased levels of Δ27bpMSH3 upon etoposide treatment (Figure 7A). With nuclear-cytosolic fractionation, Δ27bpMSH3 increased in the cytosol after etoposide, with less of an increase with the addition of MG132 to etoposide (Figure 7B, *left panel*). MG132 alone did not affect Δ27bpMSH3 protein distribution, and thus MG132 may have stabilized more nuclear than cytoplasmic Δ27bpMSH3. Interestingly, etoposide did not affect the nuclear-cytosolic distribution of NEMO/IKKγ, while MG132 did (increased the percentage of nuclear NEMO/IKKγ; Figure 7B, *right panel*). Our results indicate that ultimate reduction of total Δ27bpMSH3 protein is due to decreased MSH3 mRNA and active protein degradation upon longer etoposide treatment.

### CRM-1 Pathway for Nuclear Export Does Not Play a Role in the Distribution of Δ27bpMSH3 or NEMO/IKKγ after Induced DSBs

DNA damage triggers NEMO/IKKγ shuttling between the nucleus and cytoplasm as a part of DNA Damage Response, and the CRM-1 pathway was responsible for NEMO/IKKγ nuclear export during normal shuttling in some studies^29^ but not in others.^30^ To address if the CRM-1 pathway plays a role for transport in our experiments, we treated SW480^Δ27/Δ27^ cells with 10 nM leptomycin B (LMB), a CRM-1 inhibitor, alone and/or in combination with etoposide followed by nuclear-cytoplasmic fractionation. LMB treatment failed to alter the percentages of nuclear Δ27bpMSH3 with and/or without etoposide treatment (Figure A9). Additionally, etoposide appeared to expel NEMO/IKKγ out from the nucleus, although it did not reach statistical significance (*P* = .0587 vs control), and the addition of LMB with and/or without etoposide did not significantly affect the distribution of NEMO/IKKγ. These results indicate that CRM-1 pathway is not likely involved in the export of Δ27bpMSH3 and/or NEMO/IKKγ under our experiment conditions.

### Etoposide Treatment Increases Intracellular Reactive Oxygen Species (ROS) in Δ27bpMSH3 Cells but May Not Drive Growth Suppression as Much as Induced DSBs

We have previously demonstrated that Δ27bpMSH3 accumulates in the cytoplasm under inflammation and oxidative stress conditions.^13^ With our observation that Δ27bpMSH3 also accumulates in the cytoplasm after etoposide induces DSBs, we examined if etoposide/DSBs was a driver generating higher oxidative stress. To this end, we measured the intracellular reactive oxygen species (ROS) generation in respond to etoposide treatment. We observed increased amounts of ROS generation in a dosage-dependent manner in SW480^Δ27/Δ27^ cells, but not in CaCO2^WT/WT^ cells (Figure 7C). To help differentiate if ROS generation is as important as DSBs in prohibiting cell growth in SW480^Δ27/Δ27^ cells, we treated CaCO2^WT/WT^ and SW480^Δ27/Δ27^ cells with 50 μM H_2_O_2_ and assessed cell growth via colony formation assays. CaCO2^WT/WT^ cells were not able to form colonies that were big enough to be observed and counted, while about half of SW480^Δ27/Δ27^ colonies were present compared to control (Figure 7D). This observation suggests that SW480^Δ27/Δ27^ cells can tolerate oxidative stress markedly better than CaCO2^WT/WT^ cells, and most likely that unrepaired DSBs are a bigger driver for SW480^Δ27/Δ27^ growth suppression. Differences in ROS tolerance between cell lines are unlikely via etoposide-induced triggering of proinflammatory IL6 signaling or DSBs since both cell lines experience them via etoposide with CaCO2^WT/WT^ forming more RAD51 foci, and more likely based on each cell line’s redox handling capacity.

## Discussion

We have previously demonstrated that MSH3 reversibly shuttles from the nucleus to the cytosol under conditions of IL6-driven inflammation and oxidative stress.^6^^,^^12^^,^^13^ These previous findings indicated that inflammation and oxidative stress create a somatic loss-of-function for MSH3, relocating MSH3 to the cytosol where it could not repair dinucleotide or longer microsatellite sequence slippages at the time of DNA replication via MMR in the nucleus, nor participate in any DSB repair via HR. Loss of MSH3 function is observed in 50% of colorectal cancers as determined by its failed MMR function (EMAST), but also the accumulation of loss of heterozygosity events potentially driven by failed DSB repair at targeted sites in the genome.^31^ We also previously recognized a polymorphic form of MSH3 when we mapped the NLS of MSH3, termed Δ27bpMSH3.^13^^,^^14^ The Δ27bpMSH3 form of MSH3 has an imperfect deletion of 27 base pairs proximate to the NLS, rendering it less likely to incorporate back into the nucleus once shifted into the cytoplasm.^13^ The Δ27bpMSH3 polymorphism was predicted to be rare among humans, but examination of cell lines and human tissues suggest it might be selected for somatically.^13^^,^^14^ Here, we examined if induced DSBs by etoposide influenced MSH3 function in the absence of proinflammatory IL6. We observed that (a) Δ27bpMSH3 cells are much more susceptible to growth inhibition with induced DSBs than WT MSH3 cells but are more tolerant of exogenous oxidative stress; (b) Δ27bpMSH3 shuttles out of the nucleus and concentrates in the cytosol after induced DSBs, where it cannot help initiate DSB repair, and is done in parallel with increases in ROS; (c) the novel finding that Δ27bpMSH3 and WT MSH3 bind NEMO/IKKγ after induced DSBs within the nucleus; likely important for the DSB repair process and/or stabilization of MSH3; (d) steady state levels of available cytosolic Δ27bpMSH3 are from existing protein and new protein synthesis as well as influences from mRNA synthesis and protein degradation pathways, and nuclear-to-cytosolic shifts are not conducted via CRM-1. Our cumulative findings indicate that a nuclear-to-cytosolic shift for MSH3 makes cells more susceptible to further DNA damage after induced DSBs. Our findings also suggest that any person carrying a germline Δ27bpMSH3 allele or cell selected for the Δ27bpMSH3 allele is likely more susceptible to carcinogenesis.

We modified the protocol outlined in Dietlan et al ^25^ to allow for longer exposure to etoposide after initially not detecting any nuclear-to-cytosolic shift for Δ27bpMSH3. We became curious after Dietlan et al suggested that cells null for MSH3 or containing Δ27bpMSH3 were deficient in DSB repair (and susceptible to DNA-PK inhibitors) due to lack of Rad51 loci formation. Through our experiments, we now know the rationale for Δ27bpMSH3 being unable to participate in DSB repair is due to its novel location in the cell cytoplasm and not specifically from incapability of triggering Rad51 loci (and eliciting repair) if it were in the nucleus, meaning that nuclear Δ27bpMSH3 might function just as normal as WT MSH3. We also demonstrated that addition of a DNA-PK inhibitor did not have further effects in WT MSH3 or Δ27bpMSH3 cells indicting no differences in NHEJ repair using our protocol. This is consistent with Hinrichsen *et al.*^27^ who reported colorectal cancer cell lines lacking *MLH1* exhibited cytotoxic response to the DNA-PK inhibitor KU60648.

The issue of (nuclear) MMR and DSB repair functionality of Δ27bpMSH3 has not been fully evaluated. The MMR complex MutSβ (containing both MSH2 and MSH3) plays a key role at trinucleotide repeat expansions, integral to many neurodegenerative diseases. For instance, Huntington’s chorea patients who carry homozygous *Δ27bpMSH3* alleles have lower progression scores, later disease onsets, and lower rates of somatic trinucleotide expansions. Similarly, myotonic dystrophy type 1 patients who had biallelic *Δ27bpMSH3* also had later disease onset and lower rates of somatic trinucleotide expansions.^32^ These results are in line with our findings that Δ27bpMSH3 is not in the nucleus to adjust trinucleotide repeat lengths in the setting of conditions that are complicated with inflammation and oxidative stress. However, it remains unclear on the functionality of Δ27bpMSH3 as compared to WT MSH3 if it were located in the nucleus. The presumption is that Δ27bpMSH3 is as functional as WT MSH3 as the polymorphic features of Δ27bpMSH3 lie in its NLS site and not in MSH3’s binding and signaling sites.

NEMO/IKKγ is the modulating subunit of IKK complex essential for activation of NFκB pathway and can shuttle between the nucleus and cytosol. We identified NEMO/IKKγ as a binding partner for MSH3 in this study in addition to its normal obligate binding and stabilizing partner MSH2 (forming MutSβ). It is clear that NEMO/IKKγ-MSH3 and NEMO/IKKγ-Δ27bpMSH3 interactions occur in the nucleus after induced DSBs, but the combined complex itself does not shuttle together from the nucleus to the cytosol. Indeed, we observed that the Δ27bpMSH3 interaction with NEMO/IKKγ in the nucleus diminished over time as more Δ27bpMSH3 accumulated in the cytosol. There are a number of hypotheses for this interaction after induced DSBs. NEMO/IKKγ may be a “handoff” protein to maintain MSH3 stability after separating from MSH2 due to a stimulus such as induced DSBs. Such a combined NEMO/IKKγ-MSH3 complex might be temporary to allow MSH3 to then interact with other proteins involved in HR or be handed off to a nuclear-to-cytoplasmic transport process. Another possibility is that the NEMO/IKKγ-MSH3 interaction is a result of ROS or oxidative stress in order to either stabilize or have viable MSH3 molecules available for a variety of potential DNA damage including DSB repair, compartmentalizing and separating it from its MMR function with MSH2. Another possibility is that NEMO/IKKγ interacts with MSH3 as a sensor function, triggering or participating in other downstream inflammatory signaling processes for the cell. How the interaction between MSH3 and NEMO/IKKγ modifies cell function is not fully clear yet but certainly might play some role in the DNA Damage Response of the cell.

## Conclusion

We discovered that induced DSBs via etoposide triggers a nuclear-to-cytosolic shift for Δ27bpMSH3, removing it from the obvious site for DSB repair (and MMR) in the nucleus. WT MSH3 remains in the nucleus to be effectual in repair of DSBs. Under induced DSBs, both Δ27bpMSH3 and WT MSH3 bind NEMO/IKKγ in the nucleus, a novel finding of interaction for MSH3 and outside of its known heterodimer partner for MMR, MSH2, but does not translocate to the cytosol as a complex. This interaction might be part of the DNA Damage Response pathway but the mechanistic rationale for the NEMO/IKKγ-MSH3 and NEMO/IKKγ-Δ27bpMSH3 interactions in the nucleus are not yet fully clear. The nuclear-to-cytosolic shift for Δ27bpMSH3 makes cells more susceptible to further DNA damage after induced DSBs. Persons carrying a germline *Δ27bpMSH3* allele or cells selected for the *Δ27bpMSH3* allele are likely more susceptible to carcinogenesis from unrepaired DNA damage.

## References

[1] Carethers J.M. (2016). Hereditary, sporadic and metastatic colorectal cancers are commonly driven by specific spectrums of defective DNA mismatch repair components. Trans Am Clin Climatol Assoc.

[2] Carethers J.M., Koi M., Tseng-Rogenski S. (2015). EMAST is a form of microsatellite instability that is initiated by inflammation and modulates colorectal cancer progression. Genes.

[3] Grady W.M., Carethers J.M. (2008). Genomic and epigenetic instability in colorectal cancer pathogenesis. Gastroenterol.

[4] Boland C.R., Goel A. (2010). Microsatellite instability in colorectal cancer. Gastroenterol.

[5] Raeker M.O., Pierre-Charles J., Carethers J.M. (2020). Tetranucleotide microsatellite mutational behavior assessed in real time: implications for future microsatellite panels. Cell Mol Gastroenterol Hepatol.

[6] Tseng-Rogenski S., Chung H., Wilk M.B. (2012). Oxidative stress induces nuclear-to-cytosolic shift of MSH3, a potential mechanism for EMAST in colorectal cancer cells. PLoS One.

[7] Carethers J.M. (2017). Microsatellite instability pathway and EMAST in colorectal cancer. Curr Colorectal Cancer Rep.

[8] Carethers J.M. (2014). Differentiating Lynch-like from Lynch syndrome. Gastroenterol.

[9] Carethers J.M., Jung B.H. (2015). Genetics and genetic biomarkers in sporadic colorectal cancer. Gastroenterology.

[10] Adam R., Spier I., Zhao B. (2016). Exome sequencing identifies biallelic *MSH3* germline mutations as a recessive subtype of colorectal adenomatous polyposis. Am J Hum Genet.

[11] Koi M., Leach B.H., McGee S. (2024). Compound heterozygous *MSH3* germline variants and associated tumor somatic DNA mismatch repair dysfunction. NPJ Precis Oncol.

[12] Tseng-Rogenski S., Hamaya Y., Choi D.Y. (2015). Interleukin-6 alters localization of hMSH3, leading to DNA mismatch repair defects in colorectal cancer cells. Gastroenterol.

[13] Tseng-Rogenski S., Munakata K., Choi D.Y. (2020). The human DNA mismatch repair protein MSH3 contains nuclear localization and export signals that enable nuclear-cytosolic shuttling in response to inflammation. Mol Cell Biol.

[14] Munakata K., Koi M., Kitajima T. (2019). Inflammation-associated microsatellite alterations caused by MSH3 dysfunction are prevalent in ulcerative colitis and increase with neoplastic advancement. Clin Transl Gastroenterol.

[15] Devaraj B., Lee A., Cabrera B.L. (2010). Relationship of EMAST and microsatellite instability among patients with rectal cancer. J Gastrointest Surg.

[16] Koi M., Tseng-Rogenski S.S., Carethers J.M. (2018). Inflammation-associated microsatellite alterations: mechanisms and significance in the prognosis of patients with colorectal cancer. World J Gastrointest Oncol.

[17] Haugen A.C., Goel A., Yamada K. (2008). Genetic instability caused by loss of MutS homologue 3 in human colorectal cancer. Cancer Res.

[18] Garcia M., Choi C., Kim H.R. (2012). Association between recurrent metastasis from stage II and III primary colorectal tumors and moderate microsatellite instability. Gastroenterol.

[19] Burdova K., Mihaljevic B., Sturzenegger A. (2015). The mismatch-binding factor MutSbeta can mediate ATR activation in response to DNA double-strand breaks. Mol Cell.

[20] Bennardo N., Gunn A., Cheng A. (2009). Limiting the persistence of a chromosome break diminishes its mutagenic potential. Plos Genet.

[21] Franchitto A., Pichierri P., Piergentili R. (2003). The mammalian mismatch repair protein MSH2 is required for correct MRE11 and RAD51 relocalization and for efficient cell cycle arrest induced by ionizing radiation in G2 phase. Oncogene.

[22] Pichierri P., Franchitto A., Piergentili R. (2001). Hypersensitivity to camptothecin in MSH2 deficient cells is correlated with a role for MSH2 protein in recombinational repair. Carcinogenesis.

[23] Scully R., Panday A., Elango R. (2019). DNA-double strand break repair-pathway choice in somatic mammalian cells. Nat Rev Mol Cel Biol.

[24] Oh J.M., Myung K. (2022). Crosstalk between different DNA repair pathways for DNA double strand break repairs. Mutat Res Genet Toxicol Environ Mutagen.

[25] Dietlein F., Thele L., Jokic M. (2014). A functional cancer genomics screen identifies a druggable synthetic lethal interaction between MSH3 and PRKDC. Cancer Disc.

[26] Bannister L.A., Waldman B.C., Waldman A.S. (2004). Modulation of error-prone double-strand break repair in mammalian chromosomes by DNA mismatch repair protein Mlh1. DNA Rep.

[27] Hinrichsen I., Ackermann A., Duding T. (2017). Loss of MLH1 sensitizes colon cancer cells to DNA-PKcs inhibitor KU60648. Mol Carcinogen.

[28] Israel A. (2010). The IKK complex, a central regulator of NF-κB activation. Cold Spring Harb Perspect Biol.

[29] Verma U.N., Yamamoto Y., Prajapati S. (2004). Nuclear role of IκB Kinase-γ/NF-κB essential modulator (IKKγ/NEMO) in NF-κB-dependent gene expression. J Biol Chem.

[30] Berchtold C.M., Wu Z.H., Huang T.T. (2007). Calcium-dependent regulation of NEMO nuclear export in response to genotoxic stimuli. Mol Cell Biol.

[31] Koi M., Garcia M., Choi C. (2016). Microsatellite alterations with allelic loss at 9p24.2 signify less-aggressive colorectal cancer metastasis. Gastroenterol.

[32] Flower M., Lomeikatie V., Coisi M. (2019). MSH3 modifies somatic instability and disease severity in Huntington's and myotonic dystrophy type I. Brain.

