# Supplementary material for: Double-Strand Breaks Induce Nuclear-Cytosolic Shuttling of Polymorphic DNA Mismatch Repair Protein MutS Homolog 3 and Binding to NEMO/IKKγ in Colon Cancer Cells
Source: Gastro Hep Adv. 2025 Jul 25;4(10):100756. doi: 10.1016/j.gastha.2025.100756 (PMC12450631; doi:10.1016/j.gastha.2025.100756)
Supplement: Supplementary Figures and Legends [file mmc1.pdf]

SUPPLEMENTARY FIGURES AND LEGENDS

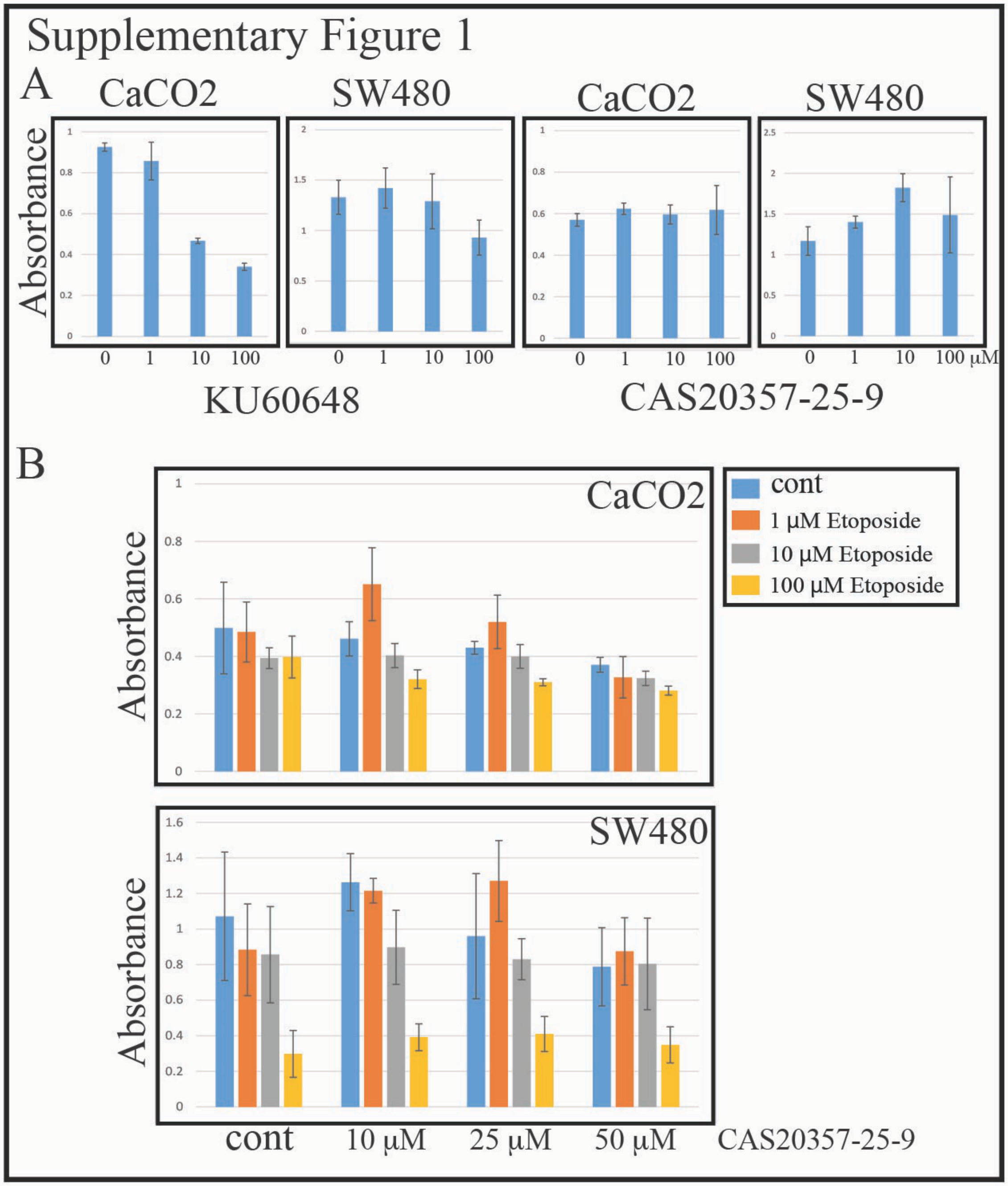

Supplementary Figure 1. SW480$^{\Delta 27/\Delta 27}$ cells carrying Δ27bpMSH3 are significantly more sensitive to etoposide but not UK60648 than CaCO2$^{WT/WT}$ cells. A. Cells were treated with increasing dosages of KU60648 and/or another DNA-PK inhibitor (CAS20357-25-9) as indicated. The proliferation assays showed that SW480$^{\Delta 27/\Delta 27}$ cells were not more sensitive to either than CaCO2$^{WT/WT}$. **B.** Cells were treated with combination of Etoposide and a DNA-PK inhibitor at various concentrations. SW480$^{\Delta 27/\Delta 27}$ cells were more sensitive to etoposide treatment than CaCO2$^{WT/WT}$. DNA-PK inhibitor did not seem to have any effects on the growth of SW480$^{\Delta 27/\Delta 27}$ cells.

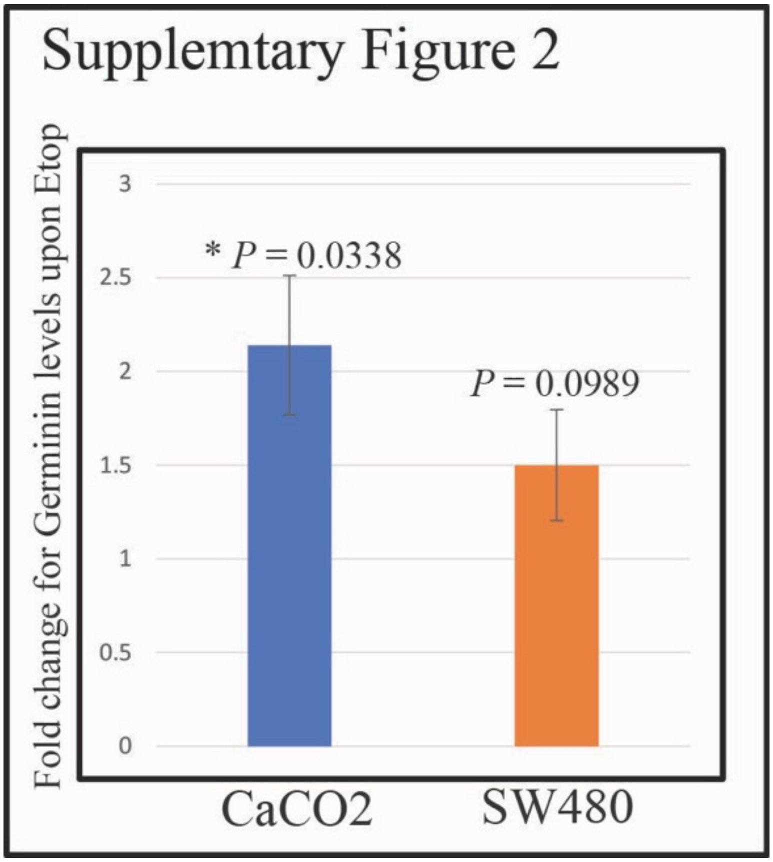

<u>Supplementary Figure 2</u>. Germinin level was significantly upregulated in CaCO2$^{WT/WT}$ but not in SW480$^{\Delta27/\Delta27}$ cells. Upon etoposide treatment, there was a significant increased expression level of Germinin in CaCO2$^{WT/WT}$, indicating cells were progressed into G2/M phase where Rad51-foci might be formed. There was a slight increase of Germinin in SW480$^{\Delta27/\Delta27}$ cells, but the increase was not statistically significant.

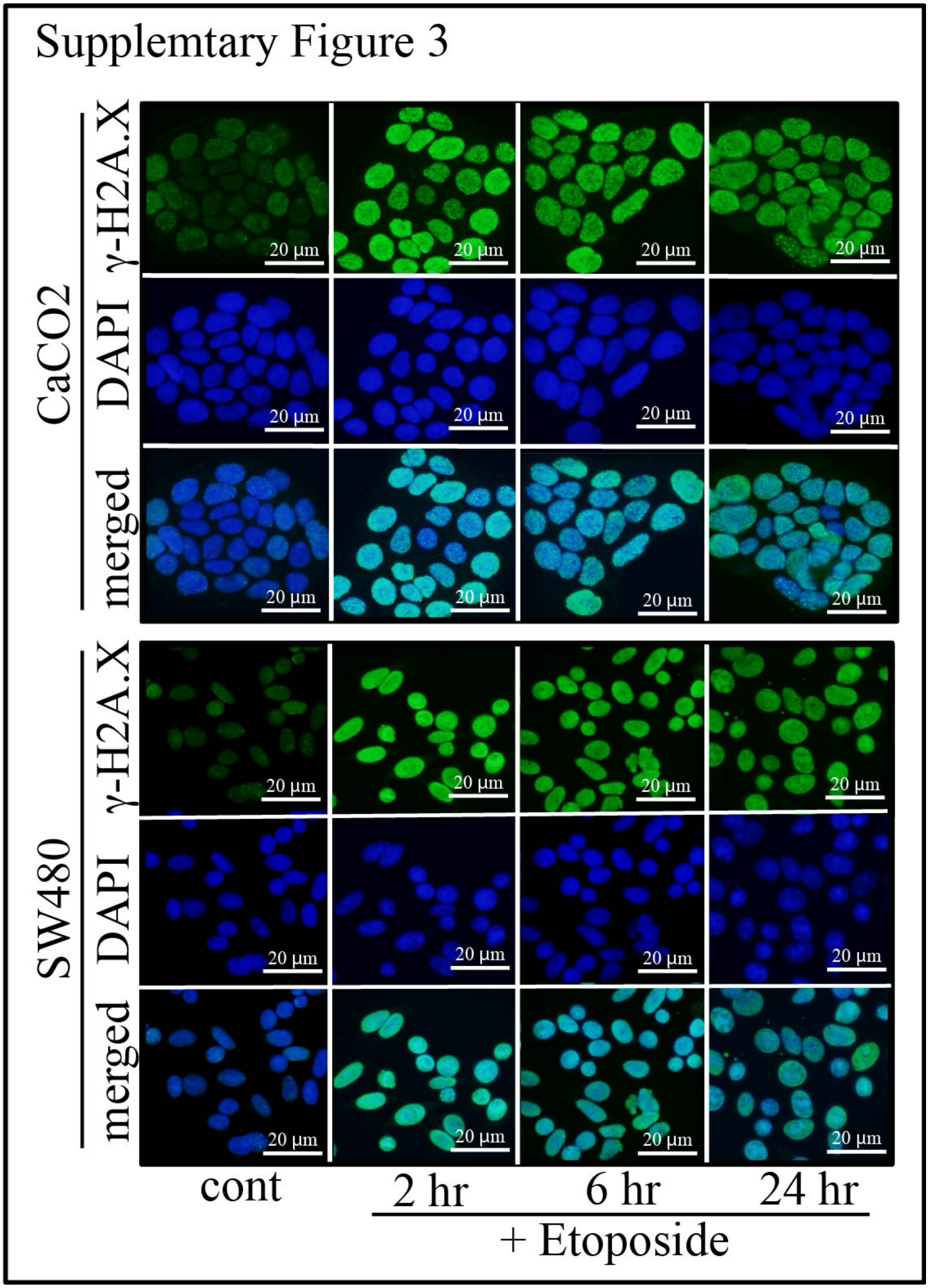

<u>Supplementary Figure 3</u>. Etoposide treatment induced upregulation of γ-H2A.X. This result demonstrated a successfully induction of DSBs in both cell lines with our treatment protocol. The time course studies indicated that the upregulation could be detected as early as 2 hours after the treatment, and it lasted for at least 24 hours. Top: CaCO2$^{WT/WT}$; bottom: SW480$^{\Delta27/\Delta27}$.

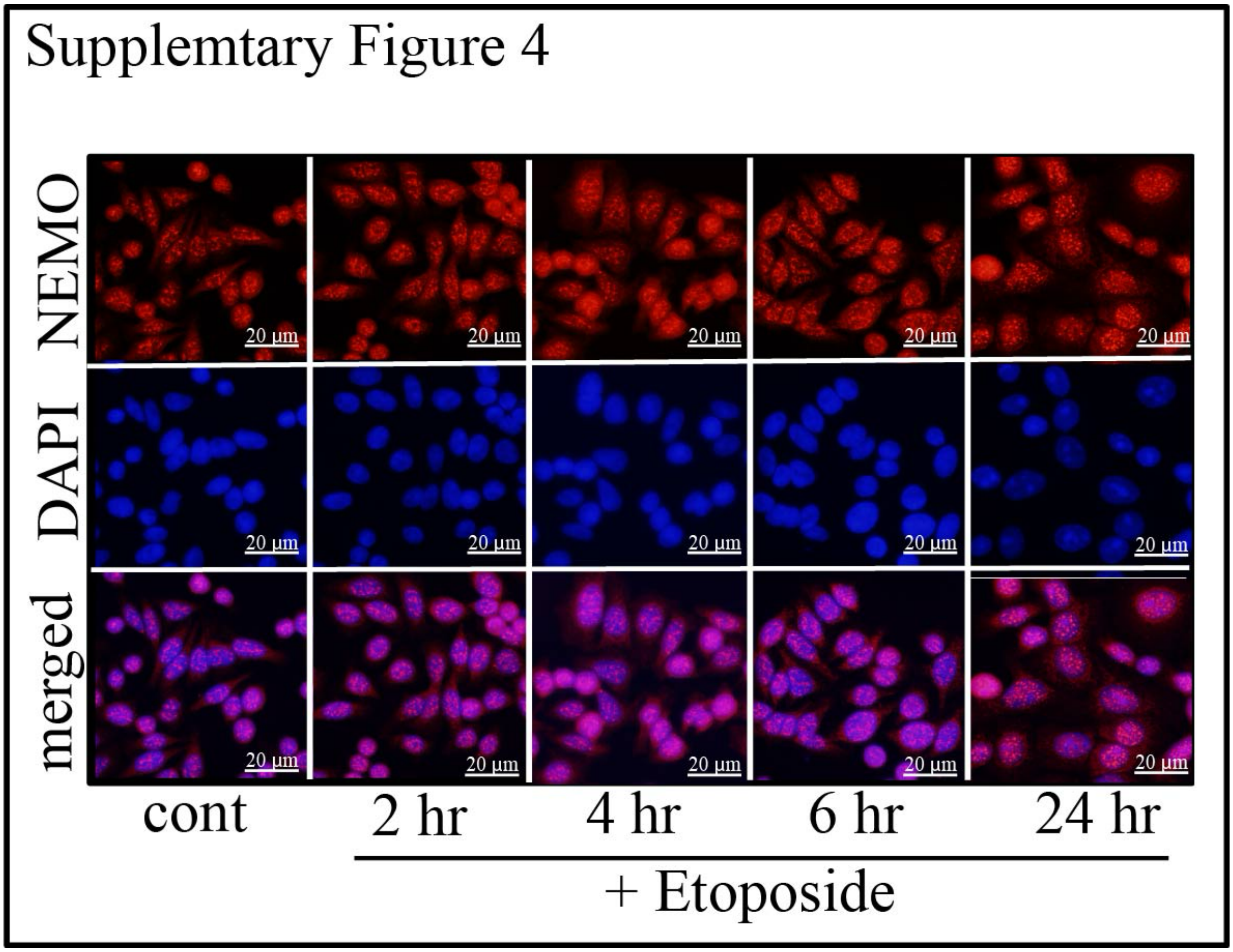

Supplementary Figure 4. NEMO/IKKγ nuclear-cytosolic distribution did not change in response to etoposide treatment. CaCO2$^{WT/WT}$ and SW480$^{\Delta 27/\Delta 27}$ cells were treated with 100 µM etoposide and stained for NEMO/IKKγ to examine its subcellular localization. IFM studies did not reveal significant changes of NEMO/IKKγ localization.

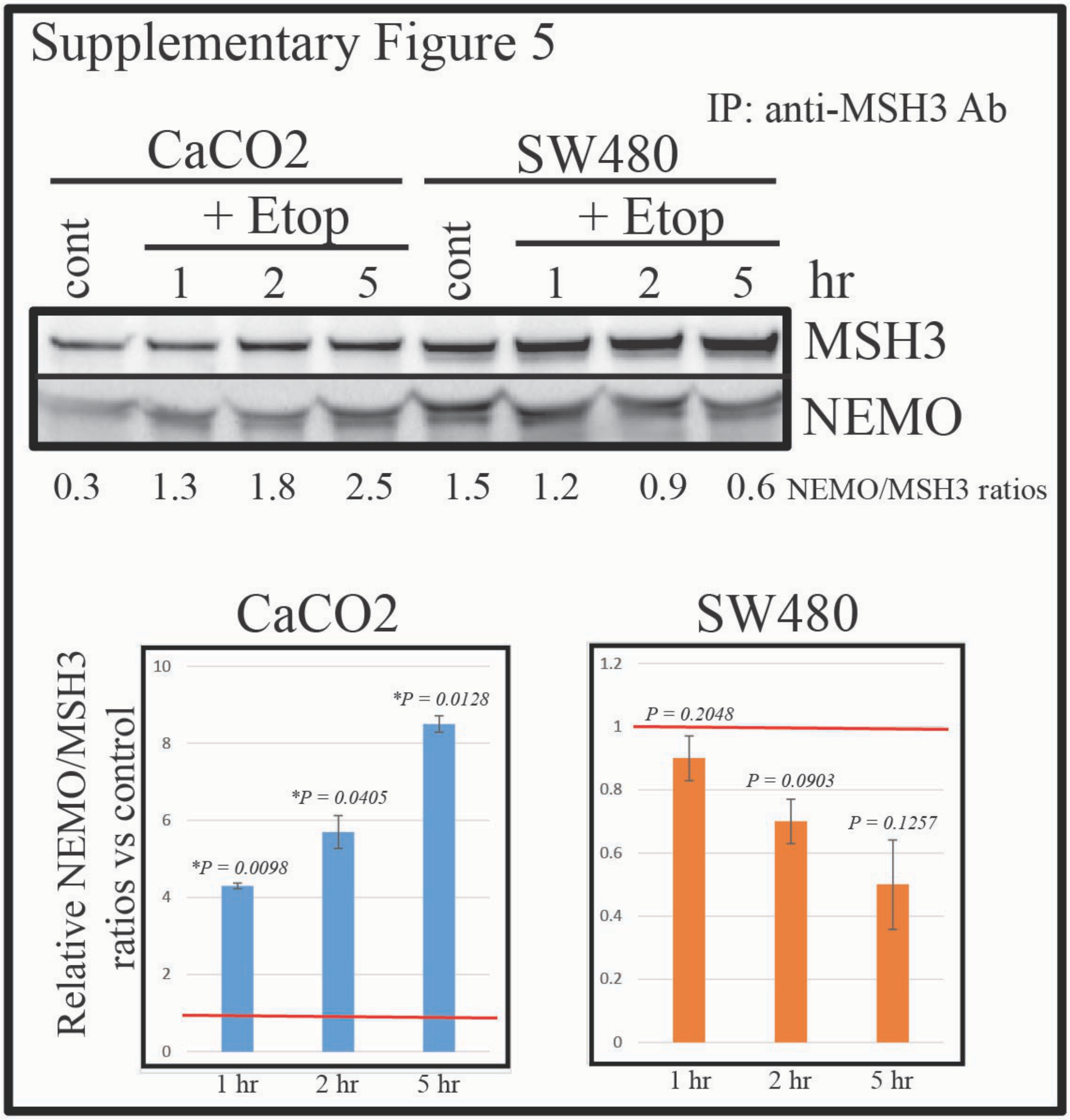

<u>Supplementary Figure 5</u>. There was an increased interaction between WTMSH3 and NEMO but decreased interaction between Δ27bpMSH3 and NEMO/IKKγ upon etoposide treatment. A time-course study of anti-MSH3 IP experiments was performed to examine the interaction between the proteins. More MSH3-NEMO interaction but less Δ27bpMSH3-NEMO/IKKγ interaction was detected as etoposide treatment proceeded longer.

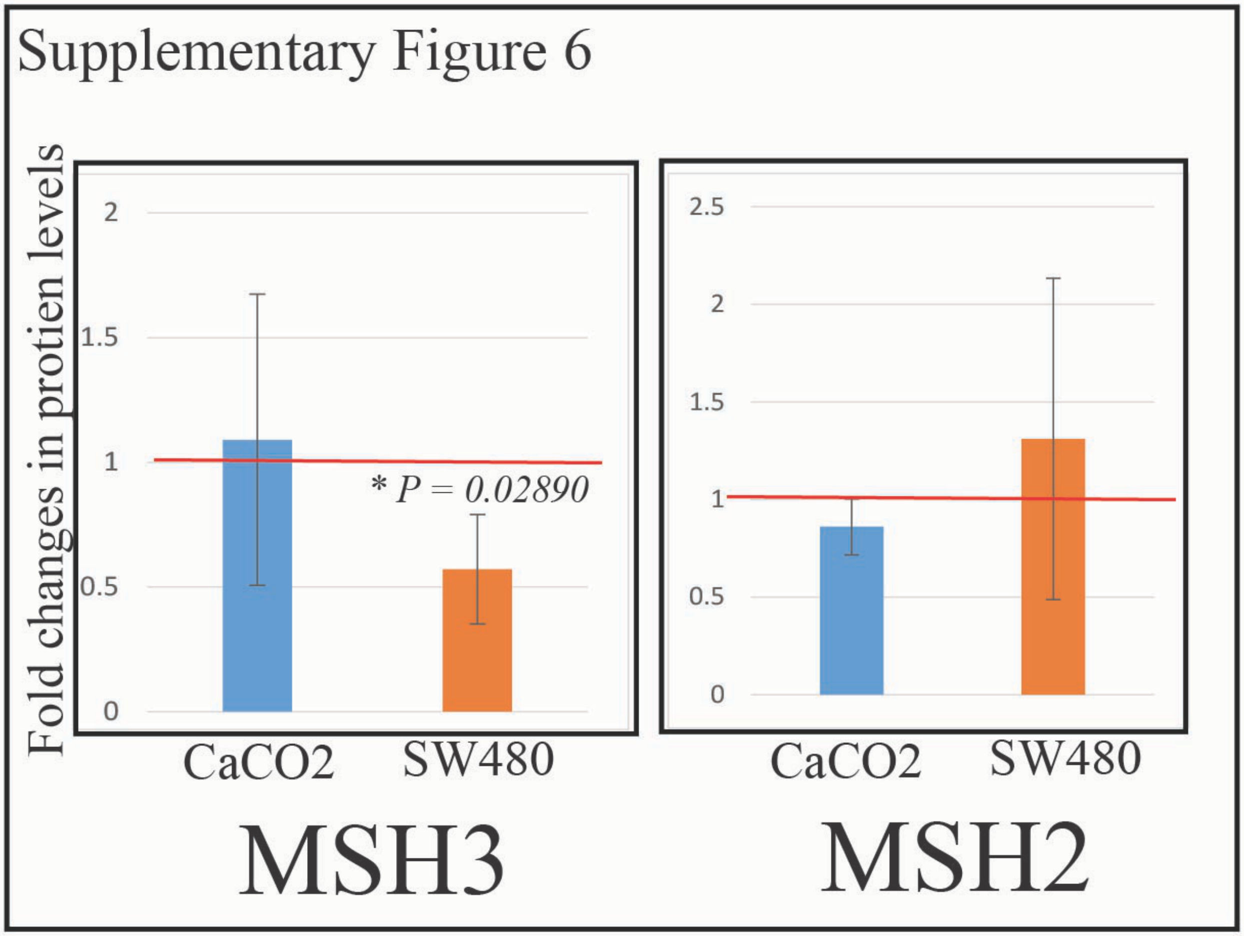

Supplementary Figure 6. WB using a second antibody confirmed the patterns of MSH3 and MSH2 level changes upon treatment. Using the same batch of the proteins, we also probed MSH3 and MSH2 using a second antibody for WB analyses. The results were consistent.

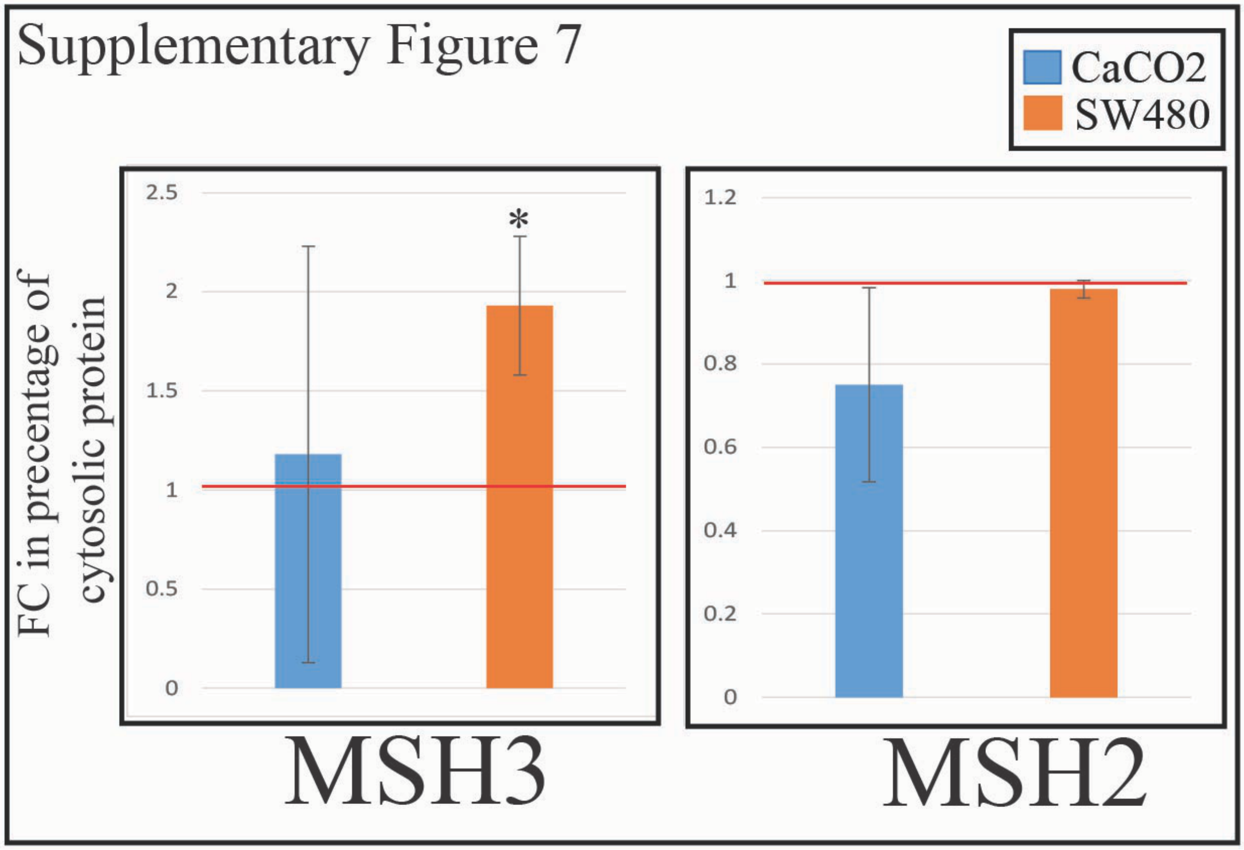

<u>Supplementary Figure 7</u>. **A second antibody confirmed the results for the nuclear-cytosolic distribution of MSH3 and MSH2.** Using the same fractionation products, we ran additional sets of WB probing he blots with a second antibody. The results were consistent with the results in Figure 8A.

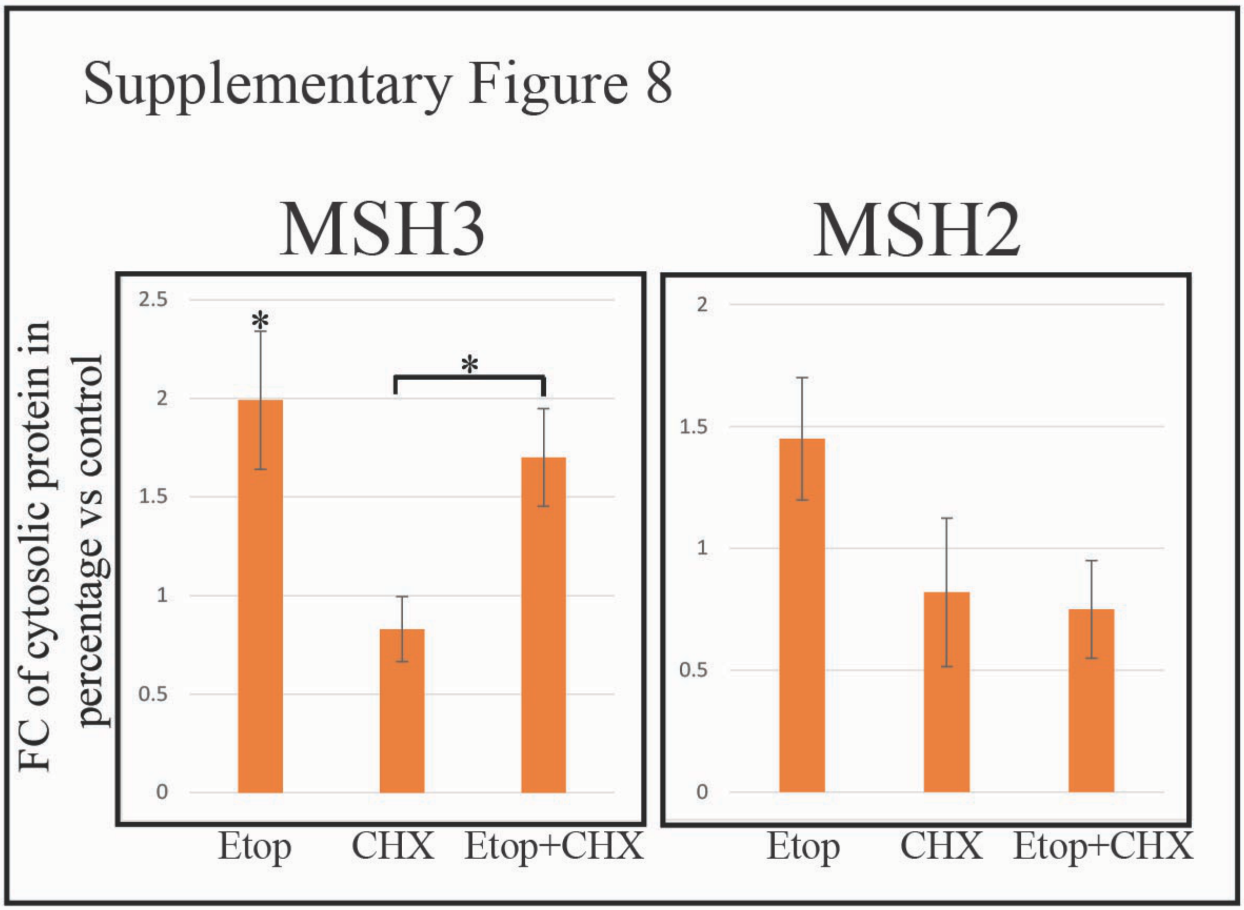

<u>Supplementary Figure 8</u>. A second set of antibodies confirmed the effects of etoposide, CHX, and/or double treatment. A second set of WB was performed using the same batch of fractionation products using different antibodies yielded the same results.

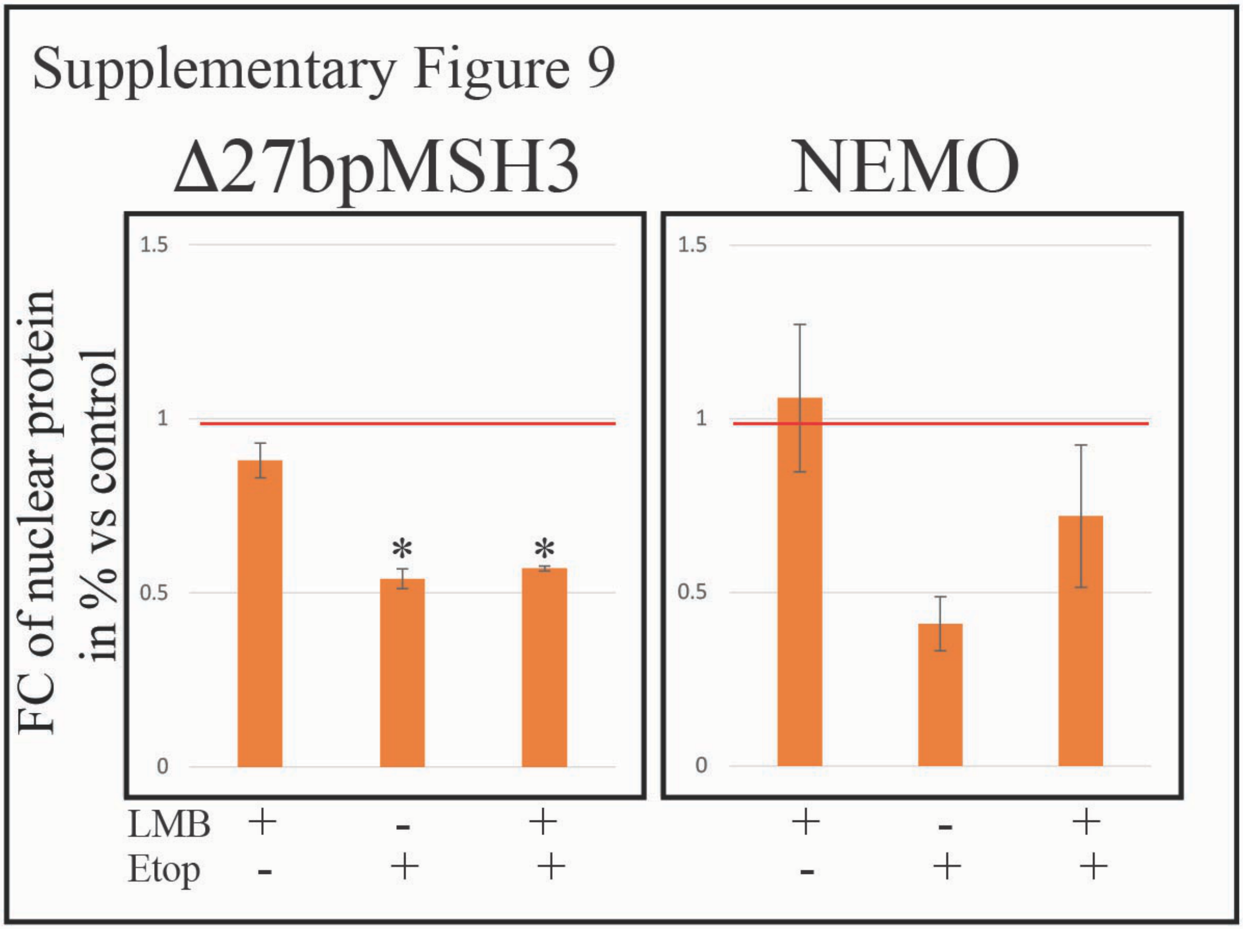

<u>Supplementary Figure 9</u>. Inhibition of CRM-1-directed nuclear export did not impact the nuclear-cytosolic distribution of NEMO/IKKγ with and/or without DNA damage, nor did it affect that of Δ27bpMSH3. SW480 cells were treated with 10 μM LMB, 100 μM etoposide, or both for 18 hr. Whole cell lysates were then fractionated into nuclear/cytosolic fractions for WB analysis. Etoposide reduced the nuclear Δ27bpMSH3 (P = 0.0277 for both etop and/or etop+ LMB vs. control). Slight nuclear NEMO/IKKγ reduction was detected upon etoposide treatment. LMB treatment did not significantly change the distribution of NEMO/IKKγ with and/or without Etoposide treatment, indicating CRM-1-directed nuclear import does not play a significant role in the NEMO/IKKγ/Δ27bpMSH3 shuttling in the experimental settings in this study.
